# Seed priming with *Streptomyces griseoviridis* culture filtrate: species-specific enhancement of legume germination and seedling vigor through hormonal and metabolic signaling

**DOI:** 10.1186/s12870-026-09261-8

**Published:** 2026-06-23

**Authors:** Rehab M. Abdelhamid, Asmaa Abdelsalam, Seifeldin Elabed, Heba El-Sayed

**Affiliations:** 1 Botany and Microbiology Department, Faculty of Science, Capital University (Formerly Helwan University), Helwan, 11795 Egypt; 2 Medical Biophysics Division, Physics Department, Faculty of Science, Capital University (Formerly Helwan University), Helwan, Egypt; 3 Biotechnology and Genetic Engineering Department, Faculty of Science, Helwan National University, Cairo, Egypt

**Keywords:** *Phaseolus vulgaris*, Photosynthetic pigments, Phytohormones, Seed priming, *Streptomyces griseoviridis*, *Vicia faba*

## Abstract

**Background:**

Actinobacteria of the genus *Streptomyces* are recognized as prolific producers of bioactive metabolites capable of regulating plant growth and stress responses. However, isolate-level phytohormone data for *Streptomyces griseoviridis*, a commercially relevant biocontrol species, remain limited and sometimes contradictory.

**Results:**

A strain of *S. griseoviridis* was isolated and molecularly characterized. High-performance liquid chromatography (HPLC) of its cell-free culture filtrate (CFF) revealed the presence of gibberellic acid (46.68 µg/100 mL), zeatin (18.90 µg/100 mL), indole-3-acetic acid (3.93 µg/100 mL), and abscisic acid (0.38 µg/100 mL), while gas chromatography-mass spectrometry (GC-MS) identified oleic acid (17.1%) and palmitic acid methyl ester (8.34%) as major metabolites. Seeds of *Phaseolus vulgaris* and *Vicia faba* were primed with varying concentrations of CFF. In *P. vulgaris*, the 25% CFF significantly enhanced germination percentage (GP; 66.7% vs. 60.0% control), germination energy (GE; 56.7 vs. 41.7), germination rate index (GRI; 4.02 vs. 3.11), stem length (20.80 vs. 16.10 cm), and total photosynthetic pigments (3.79 vs. 2.8 mg/g). In *V. faba*, the 10% CFF proved most effective, achieving 100% GP (control: 98.3%), increasing GE and GRI, reducing mean germination time, and elevating total pigments (3.11 vs. 1.86 mg/g). Regarding soluble metabolites, soluble protein content in *P. vulgaris* was maximized by the 25% and 50% CFF treatments, reaching 165.4 and 136.8 mg/g dry weight, respectively, compared to 108.9 mg/g in the control, while soluble sugars increased with 10% CFF concentrations. In *V. faba*, the highest soluble protein concentration (295.4 mg/g) was achieved at 10% CFF (control: 202.1 mg/g), whereas soluble sugars reached a maximum at 100% CFF. Molecular docking revealed compound 2-acetyl-3-(2-cinnamido)ethyl-7-methoxyindole (indole-related) exhibited strong binding to NCED1 (− 7.85 kcal/mol), CCD1 (− 9.21 kcal/mol), starch phosphorylase (− 7.03 kcal/mol), and AGPase (− 6.56 kcal/mol), outperforming controls and fatty acid derivatives.

**Conclusion:**

The culture filtrate of *Streptomyces griseoviridis* constituted a biologically active signaling pool capable of modulating legume primary metabolism and photosynthetic pigment profiles in a species- and concentration-dependent manner. This study provided novel isolate-level evidence of the hormonal and metabolic effects of *S. griseoviridis* filtrates and pointed out the potential of plant growth-promoting *Streptomyces* species as sustainable drivers of crop productivity and food security.

## Background

The growing global need for food, propelled by a swiftly increasing population, has imposed extraordinary pressure on agricultural systems to improve crop yields. Conventional methods for narrowing the yield gap have predominantly depended on synthetic chemical fertilizers and the advancement of genetically modified organisms. The prolonged use of agrochemicals is linked to soil health deterioration, bioaccumulation of persistent compounds, and negative ecological impacts, but the employment of genetically modified organisms continues to be contentious and subject to regulatory examination [1]. Therefore, there is an immediate necessity for sustainable and eco-friendly alternatives that can improve agricultural productivity without jeopardizing ecosystem integrity. In this context, microbial diversity has emerged as a promising domain, providing a substantial reservoir of unique genes and metabolic pathways for the development of natural bioproducts that can enhance plant growth and health.

Actinobacteria, particularly the species Streptomyces, stand out among soil microorganisms as prolific producers of bioactive secondary metabolites. These Gram-positive, filamentous bacteria are omnipresent in soil and are distinguished by their ability to synthesize a wide variety of chemicals, encompassing two-thirds of all known antibiotics. In addition to their established function in biocontrol, Streptomyces species are increasingly acknowledged for their plant growth-promoting (PGP) attributes [2]. They accomplish this feat via several ways, including the direct synthesis of phytohormones such as indole-3-acetic acid (IAA), gibberellins, and cytokinins, which are essential regulators of plant growth and development. Their capacity to produce durable spores renders them especially appealing as stable and effective biocontrol and biofertilizer agents for agricultural purposes [1].

The PGP potential of the genus *Streptomyces* is well recognized, but isolate-level data for the commercially important species are often limited and sometimes contradictory. *Streptomyces griseoviridis*, an actinobacterial strain employed in commercial biocontrol formulations. Notwithstanding its significance, extensive qualitative and quantitative data regarding its phytohormone biosynthetic capability are markedly limited. A previous investigation examining *S. griseoviridis* from the bean rhizosphere indicated the absence of detectable levels of common plant growth regulators, such as IAA and zeatin, in its culture filtrates [3]. This emphasizes a significant knowledge deficiency and stresses the need for strain-specific characterization to comprehensively comprehend and utilize the metabolic potential of this species for agricultural advantage.

Microbial seed priming, a novel sustainable approach, provides a direct and effective means to harness the plant growth-promoting potential of beneficial microorganisms [4]. This method entails pre-soaking seeds in a microbial culture or its cell-free filtrate (CFF) to provide a range of bioactive compounds that can improve germination, seedling vigor, and initial establishment. The regulatory effects of priming are dictated by a complex interaction of hormonal signals, including gibberellic acids that initiate reserve mobilization [5, 6] and cytokinins that facilitate cell proliferation [7], counterbalanced by inhibitory hormones such as abscisic acid (ABA) [8]. The exact effects of *S. griseoviridis* CFF on essential physiological processes—such as the accumulation of photosynthetic pigments and primary metabolism in leguminous crops are little characterized, especially regarding species-specific responses and concentration-dependent effects.

The aim of this study was to isolate and characterize a strain of *Streptomyces griseoviridis*, evaluate its potential for the production of essential phytohormones and other bioactive metabolites and assess the biostimulatory capacity of its cell-free culture filtrate as a seed priming agent for improving germination, seedling growth and physiological performance in legume crops. Furthermore, the identified metabolites were docked with plant protein targets to understand the mechanistic basis of the observed germination and pigmentation responses.

## Materials and methods

### Isolation of actinobacteria strain

An actinobacterial strain was isolated from a soil sample collected from a garden within the campus of Helwan University, Cairo, Egypt (29.8687° N, 31.3197° E).” Soil samples were air-dried for one week, crushed, and then sieved. One gram of soil was mixed with 0.1 g of calcium carbonate and incubated at 37 °C for 2–5 days. This pretreatment enhanced the population of actinobacteria [9]. Isolation of Actinobacteria was performed by serial dilution and spread plate technique using starch casein agar (soluble starch: 10 g, K_2_HPO_4_: 2 g, KNO_3_: 2 g, casein: 0.3 g, MgSO_4_.7H_2_O: 0.05 g, CaCO_3_: 0.02 g, FeSO_4_.7H_2_O: 0.01 g, agar: 15 g, in 1000 mL tap water and pH: 7.0 ± 0.1) [10]. One gram of soil sample was taken in 9 mL of distilled water and mixed properly. Serial dilution was made. The sample was inoculated in the isolation medium plates from each dilution. Tetracycline and ampicillin were added to media to inhibit bacterial contamination. Each dilution was plated in triplicate (*n* = 3). Plates were incubated at 30 °C for 2–7 days.

### Identification of the actinobacterial isolate

Phenotypic and molecular identification of the actinobacterial isolate was performed at Assuit University Mycological Center (AUMC), Cairo, Egypt. The actinobacterial isolate was cultured in sterile Petri plates containing starch casein agar medium and incubated at 28 °C for 7 days [11]. A small amount of the actinobacterial culture was suspended in 1 mL of sterile distilled water in Eppendorf tubes and boiled for 15 min. The dead actinobacterial cells (1 mL volume) were shipped to SolGent Company, Daejeon, South Korea, for DNA extraction, polymerase chain reaction (PCR), and gene sequencing.

### DNA extraction

DNA was extracted and isolated by the Solg™ Genomic DNA Prep Kit, which extracts genomic DNA using a glass microfiber membrane. It is a kit for obtaining high-quality DNA by removing PCR inhibition substances (divalent cations and proteins, etc.).

### Polymerase Chain Reaction (PCR)

PCR was performed to amplify the 16S rRNA gene using two universal primers, namely 27 F (5’-AGAGTTTGATCC TGGCTCAG-3’) and 1492R (5’-GGTTACCTTGTTA CGACTT-3’). The polymerase chain reaction (PCR) mixture was prepared using Solgent EF-Taq as follows: 10X EF-Taq buffer 2.5 µL, 10 mM dNTP (T) 0.5 µL, primer (F-10p) 1.0 µL, primer (R-10p) 1.0 µL, EF-Taq (2.5U) 0.25 µL, template 1.0 µL, and DW to 25 µL. Then the amplification was carried out using an ABI 9700 thermal cycler under the following PCR reaction conditions: one round of amplification was performed consisting of denaturation at 95 °C for 15 min followed by 30 cycles of denaturation at 95 °C for 20 s, annealing at 50 °C for 40 s, and extension at 72 °C for 1 min, with a final extension step of 72 °C for 5 min [12]. Then the PCR products were purified with the SolGent PCR Purification Kit-Ultra (SolGent, Daejeon, South Korea) prior to sequencing. The purified PCR products were reconfirmed (using a size marker) by electrophoresis on 1% agarose gel.

### Sequencing of 16 S rRNA gene

Bands were eluted and sequenced in the sense and antisense direction using the same primers with the incorporation of ddNTPs for chain termination (Sanger sequencing). Contigs were created from the sequencing data using the CLCBio Main Workbench program. The obtained sequences were further analyzed using BLAST from the National Center of Biotechnology Information (NCBI) website (https://blast.ncbi.nlm.nih.gov/Blast.cgi). Sequences obtained together with those retrieved from the GenBank database (http://www.ncbi.nlm.nih.gov) were subjected to the Clustal W analysis using MegAlign software version 5.05 (DNASTAR Inc., Madison, Wisconsin, USA) for the phylogenetic analysis [12].

### Preparation of cell-free actinobacterial filtrate

The cell-free filtrate (CFF) was prepared by inoculating *S. griseoviridis* spores, which were removed from starch casein agar and added to 50 mL of inoculation medium (starch-casein broth) in a 250 mL conical flask. The flask was then placed in a rotary shaker set at 120 rpm for 48 h at 28 °C. After that, 10% of the inoculum was added into 50 mL of sterile starch-casein broth in 250-mL Erlenmeyer flasks, followed by incubation at 30 °C with shaking at 120 rpm for 8 days. Cultures were centrifuged at 10,000 × *g* for 15 min at 4 °C, and the supernatant was sequentially sterilized through Whatman No. 1 filter paper and a 0.22-µm membrane (Millipore, USA). The sterile filtrate was designated as 100% (v/v) CFF stock.

### Extraction of phytohormones

Phytohormone extraction from the cell-free filtrate (CFF) was performed following a well-established solvent-partitioning protocol [13]. For the cell-free filtrate, centrifuge at 4.000–10.000 × g at 4 °C for 10–15 minto remove actinobacterial cells and debris. The supernatant was acidified to pH 2.5–3 with 1 M HCl, and an equal volume of ethyl acetate (El-Gomhouria Company, Cairo, Egypt) was added. The mixture was shaken vigorously for 10 min and then left overnight at 4 °C to allow complete phase separation. The organic (upper) phase was collected, and the extraction was repeated twice with fresh ethyl acetate. The pooled organic phases were dried over anhydrous sodium sulfate and concentrated to dryness using a rotary evaporator under reduced pressure at a bath temperature of 30–40 °C. The residue was resuspended in 1 mL of HPLC-grade methanol, filtered through a 0.22 μm syringe filter, and stored at − 20 °C until analysis [14].

### HPLC analysis of phytohormones

Quantitative analysis was subsequently conducted via high-performance liquid chromatography (HPLC) at the Desert Research Center (DRC), Cairo, Egypt, using standard operational protocols. Chromatographic separation was achieved using a Waters HPLC system (Waters Corporation, Milford, MA, USA) comprising a 1525 binary pump, a 2707 auto-sampler, and a 2489 UV/Vis detector. A reverse-phase C18 column (µBondapack, 300 mm × 3.9 mm i.d.; Waters Corporation, Milford, MA, USA) served as the stationary phase. The mobile phase consisted of a binary gradient of 0.1% (v/v) acetic acid in ultrapure water (solvent A) and HPLC-grade acetonitrile (solvent B), delivered at a constant flow rate of 1.0 mL min⁻¹. For detection, the system was configured for dual-wavelength monitoring: 254 nm for acidic phytohormones (indole-3-acetic acid, abscisic acid, and gibberellic acid) and 269 nm for cytokinin compounds. The phytohormone analysis was performed in triplicate (*n* = 3). Identification of target analytes was based on the congruence of their retention times with those of authenticated external standards (Sigma-Aldrich, St. Louis, MO, USA). Quantification was performed by integrating peak areas and interpolating values from linear external calibration curves constructed for each standard.

### Gas chromatography-mass spectrometry (GC-MS) of the CFF

The cell-free filtrate was subjected to GC-MS analysis. For analyses of CFF metabolites, an additional derivatization procedure was employed: dried samples (5 mg) were reacted with 100 µL of a derivatization reagent consisting of N, O-bis(trimethylsilyl) trifluoroacetamide (BSTFA) and trimethylchlorosilane (TMCS) (80:20, v/v), followed by incubation at 65 °C for 1 h. Derivatized samples were analyzed using a GC-TSQ mass spectrometer (Thermo Fisher Scientific, Austin, TX, USA) fitted with a TG–5MS capillary column (30 m × 0.25 mm i.d., 0.25 μm film thickness). Helium served as the carrier gas at a constant flow rate of 1.0 mL/min in both systems. For underivatized extracts, the oven temperature was initially held at 60 °C for 1 min, then raised at 4.0 °C/min to 240 °C and held for 1 min, with the injector maintained at 210 °C and a split ratio of 1:10. For derivatized samples, the temperature program began at 60 °C, increased at 5 °C/min to 250 °C (hold 2 min), then ramped at 30 °C/min to 300 °C, with the injector at 270 °C and injection performed in split mode using an Autosampler AS3000. Electron ionization (EI) was applied at 70 eV on both instruments. Mass spectra were acquired in full-scan mode, covering *m/z* range of 50–650 for derivatized analysis. The ion source temperature was set to 210 °C for the ISQ system and 200 °C for the TSQ system, while the transfer line temperature for the TSQ was maintained at 280 °C. The GC-MS analysis was carried out in triplicate (*n* = 3).

### Plant materials

Seeds of *Vicia faba* and *Phaseolus vulgaris* were procured from the Crop Institute at the Agricultural Research Center, located in Giza, Egypt.

### Priming treatment and germination assay

Seeds of *Vicia faba* and *Phaseolus vulgaris* were surface sterilized by immersion in 1.05% (v/v) sodium hypochlorite solution for 10 min, followed by three thorough rinses with sterile distilled water. Priming was performed by immersing the seeds for 6 h in cell-free filtrate (CFF) solutions at concentrations of 10%, 25%, 50%, and 100% (v/v) using a ratio of 5 mL CFF per gram of seeds. Seeds immersed in sterile distilled water served as the negative control. After priming, seeds were rinsed with distilled water and placed in 9 cm diameter Petri dishes lined with Whatman No. 2 filter paper. The experiment was arranged in a completely randomized design with six replicates per treatment, each replicate consisting of one Petri dish containing five seeds. Dishes were incubated in a growth chamber at 25 ± 2 °C under cool white fluorescent light (3000 lx) with an 8-h photoperiod. Germination was monitored daily for six days; a seed was considered germinated upon visible radicle emergence. The following germination parameters were calculated:

$$\begin{aligned}&Germination\:Percentage\:\left(GP\:\%\right)\\&=\left(\frac{Total\:number\:of\:germinated\:seeds\:}{\:Total\:number\:of\:seeds}\right)\times\:\:100\end{aligned}$$

$$\begin{aligned}&Germination\:Energy\:\left(GE\:\%\right)\:=\\&\left(\frac{Number\:of\:germinated\:seeds\:on\:day\:4\:}{\:Total\:number\:of\:seeds}\right)\times\:\:100\end{aligned}$$

$$\:Germination\:Rate\:Index\:\left(GRI\right)\:=\:\varSigma\:\left(\frac{Gt\:}{\:Tt}\right)$$

where Gt is the number of seeds germinated on day *t*, and Tt is the corresponding day.

$$\begin{aligned}&Mean\;Germination\;Time\:\left(MGT\right)\\&=\frac{\varSigma\:\:(Ti\:\times\:\:Ni)\:}{\:\varSigma\:\:Ni}\end{aligned}$$

where Ni is the number of seeds newly germinated at time Ti.

### Plant cultivation and experimental design

After seven days of germination on filter paper, uniformly sized seedlings were selected and transplanted into plastic pots under controlled growth conditions. Each pot was filled with homogenized soil containing 55% clay, 26% silt, and 19% sand, characterized by a pH of 7.5, electrical conductivity of 2.5 dS m⁻¹, calcium carbonate content of 5.3%, and organic matter content of 1.0%. The soil contained nitrogen (135.0 mg kg⁻¹), phosphorus (115.6 mg kg⁻¹), potassium (122.0 mg kg⁻¹), calcium (148.0 mg kg⁻¹), and magnesium (119.0 mg kg⁻¹). Prior to pot filling, the soil was thoroughly homogenized and uniformly distributed among treatments to ensure consistent physicochemical conditions across all experimental units. The experiment was arranged in a completely randomized design with six pots per treatment, each pot containing five seedlings spaced approximately 1.0 cm apart. The entire experiment was independently repeated three times.

Four weeks after planting, growth parameters were recorded. Seedlings were then oven-dried at 50 °C for 72 h to determine dry weight. Dry weights of leaves, stems and roots were recorded separately. Leaf area was determined using the length-width method with shape-specific correction factors: 0.66 for V. faba and 0.97 for P. vulgaris, according to the following equations:

$$\begin{aligned}&Vicia\:faba\:leaf\:area\:(cm^2)\\&=\:(leaf\:length\:\times\:\:leaf\:width)\times\:\:0.66\end{aligned}$$

$$\begin{aligned}&Phaseolus\:vulgaris\:leaf\:area\:(cm^2)\:\\&=\:(leaf\:length\:\times\:\:leaf\:width)\times\:\:0.97\end{aligned}$$

### Pigment analysis

Chlorophyll a, chlorophyll b and total carotenoid contents were quantified spectrophotometrically according to Lichtenthaler and Buschmann [15]. Fresh leaf tissue (0.5 g) was homogenized in 5 mL of 85% (v/v) acetone and centrifuged at 10,000 rpm for 15 min. The supernatant was collected and the volume adjusted to 20 mL with 85% acetone. Absorbance of the extract was measured at 452, 645 and 664 nm using a UV/VIS Helios Gamma spectrophotometer, with 85% acetone as the blank. Pigment concentrations were calculated using the following equations:

$$\begin{aligned}Chlorophyll\:a\:(\mu\:g/mL)\:&=(10.3\:\times\:\:A_{664})\\&-(0.918\:\times\:\:A_{645})\end{aligned}$$

$$\begin{aligned}Chlorophyll\:b\:(\mu\:g/mL)&=\:(19.7\:\times\:\:A_{645})\\&-(3.87\:\times\:\:A_{664})\end{aligned}$$

$$\begin{aligned}Carotenoids\:(\mu\:g/mL)&=\:(4.3\:\times\:\:A_{452})\\&-\:\left[0.0265\right(Chl\:a)\\&+\:0.426(Chl\:b\left)\right]\end{aligned}$$

### Biochemical analyses

#### Metabolites extraction

Fresh leaf tissue (0.1 g) was homogenized in 5 mL of 70% (v/v) aqueous ethanol. The homogenate was centrifuged, and the supernatant was collected and adjusted to 15 mL with distilled water. This extract was used for subsequent determination of total soluble sugars and total soluble proteins.

#### Determination of total soluble sugars

Total soluble sugars were quantified using the anthrone-sulfuric acid method as described by Umbreit et al. [16]. Briefly, 3 mL of the ethanolic extract was mixed with 6 mL of anthrone reagent (2 g L⁻¹ in 95% sulfuric acid). The mixture was heated in a boiling water bath for 3 min, then cooled to room temperature. Absorbance of the resulting green color was measured at 620 nm using a spectrophotometer.

#### Determination of total soluble proteins

Soluble protein content was determined according to Lowry et al. [17] with modifications for plant leaf tissue. Leaf samples (0.5 g) were homogenized in 5 mL of ice-cold 0.1 M phosphate buffer (pH 7.0) and centrifuged at 12,000 × g for 20 min at 4 °C. To remove interfering phenolic compounds, 1 mL of the supernatant was mixed with an equal volume of ice-cold 10% (w/v) trichloroacetic acid (TCA) and incubated on ice for 30 min. After centrifugation at 10,000 × g for 10 min, the supernatant was discarded, and the protein pellet was redissolved in 1 mL of 0.1 N NaOH. An aliquot (0.2 mL) of this purified extract was brought to 1 mL with distilled water, then mixed with 5 mL of alkaline copper reagent (freshly prepared 50:1 v/v mixture of 2% Na₂CO₃ in 0.1 N NaOH and 0.5% CuSO₄·5 H₂O in 1% sodium potassium tartrate). After 10 min, 0.5 mL of Folin-Ciocalteu phenol reagent (diluted 1:3 with distilled water) was added rapidly with immediate mixing. The samples were incubated in the dark for 30 min, and absorbance was measured at 750 nm using a spectrophotometer. Bovine serum albumin (BSA) was used as a standard. Soluble protein content was expressed as mg per gram dry weight after determining the fresh-to-dry weight ratio from a separate subsample dried at 80 °C to constant weight. All measurements were performed in triplicate.

### Molecular docking study

The *S. griseoviridis* metabolites were evaluated for bioactivity on seed germination and pigmentation-related traits in two plant species, *Vicia faba* L. (faba bean) and *Phaseolus vulgaris* L. (common bean). Four primary targets were selected based on their functional relevance to the observed phenotypes: 9-cis-epoxycarotenoid dioxygenase (NCED1, Q9M6E8, *Phaseolus vulgaris*), which catalyzes the committed first step of abscisic acid (ABA) biosynthesis and is directly linked to seed dormancy and germination control; carotenoid cleavage dioxygenase 1 (CCD1, Q94IR2, *Phaseolus vulgaris*), which catalyzes carotenoid turnover and apocarotenoid-derived pigment homeostasis; plastidial starch phosphorylase (Pho1, P53536, *Vicia faba*), a pyridoxal-phosphate-dependent glucan-processing enzyme essential for carbohydrate mobilization during early seed development; and ADP-glucose pyrophosphorylase small subunit (AGPase-S, P52416, *Vicia faba*), which catalyzes the committed step of ADP-glucose synthesis and is rate-limiting for starch synthesis. This selection was justified by two convergent functional axes: ABA signaling and carotenoid metabolism (NCED1 and CCD1) regulate germination and pigmentation, whereas starch-metabolism enzymes (Pho1 and AGPase) govern carbohydrate availability for seedling energetics. Domain architecture and catalytic-site validity for all four targets were confirmed using UniProt (https://www.uniprot.org/) and InterProScan annotations, which verified the presence of conserved catalytic motifs and supported the functional classification of each target [18, 19].

Protein structures were obtained from the AlphaFold Protein Structure Database and used as high-confidence predicted models [20]. The four primary targets were modeled as follows: Q9M6E8 (NCED1, *Phaseolus vulgaris*) from an AlphaFold structure with the catalytic center defined by conserved iron-coordinating geometry; Q94IR2 (CCD1, *Phaseolus vulgaris*) from an AlphaFold prediction; P53536 (Pho1, *Vicia faba*) from an AlphaFold structure; and P52416 (AGPase-S, *Vicia faba*) from an AlphaFold prediction. Receptor preparation followed standard protocols: crystallographic water molecules were removed; alternate conformations were resolved by selecting the highest-occupancy state; co-crystallized inhibitors or products were deleted to allow unbiased docking into the free pocket; and all structures were checked for completeness and stereochemical validity. Polar hydrogens and Gasteiger partial charges were assigned using AutoDockTools [21]. For each receptor, the catalytic or functional site was defined on the basis of either experimentally validated ligand-binding coordinates or conserved catalytic motifs identified from sequence annotations. In particular, for the two carotenoid oxygenases (NCED1 and CCD1), the docking box was centered on the conserved histidine clusters coordinating the catalytic iron center; for Pho1, the box encompassed the extended catalytic channel containing the pyridoxal-phosphate binding pocket and glucan-processing groove; and for AGPase, the box covered the nucleotidyl-transferase catalytic cleft in the N-terminal domain. Grid dimensions ranged from 22 to 34 Å per side, depending on target geometry and ligand size, to ensure comprehensive coverage of the catalytic environment without introducing excessive noise into the docking score.

Ligand preparation included 17 compounds (ED00–ED16), generated from SMILES strings using Open Babel [22]. Structures were energy-minimized with the MMFF94 force field, and rotatable bonds were defined automatically. Docking was performed using GNINA, which integrates classical scoring with convolutional neural network-based rescoring [23]. For each ligand–target pair, 10 independent docking runs were executed with exhaustiveness values of 16–24 depending on binding-site size. The top 10 poses were retained to calculate mean binding affinity (ΔG, kcal/mol) and standard deviation, and the best-ranked pose for each ligand–target pair was selected for downstream analysis. Interaction profiling was conducted using PLIP, enabling systematic identification of hydrogen bonds, hydrophobic contacts, salt bridges, and π- stacking interactions [24]. Quantitative geometric thresholds were applied to ensure mechanistic validity: hydrogen bonds were considered strong when donor–acceptor distance was < 3.2 Å and angle was > 120° [25]; salt bridges were defined at distances < 4.5 Å between charged residues [26]; hydrophobic interactions were considered significant within 3.4–4.0 Å [27]; and π- stacking interactions were validated when centroid distances were < 5.5 Å and angular deviation was < 30° [28].

### Statistical analysis

All germination and growth data were subjected to analysis of variance (ANOVA). Means from six independent replicates per treatment were compared using Tukey’s honestly significant difference (HSD) post-hoc test at a significance level of *p* ≤ 0.05, implemented in Minitab software. Data visualization was performed using R version 4.2.2.

## Results

### Isolation and molecular characterization

**Fig. 1:**
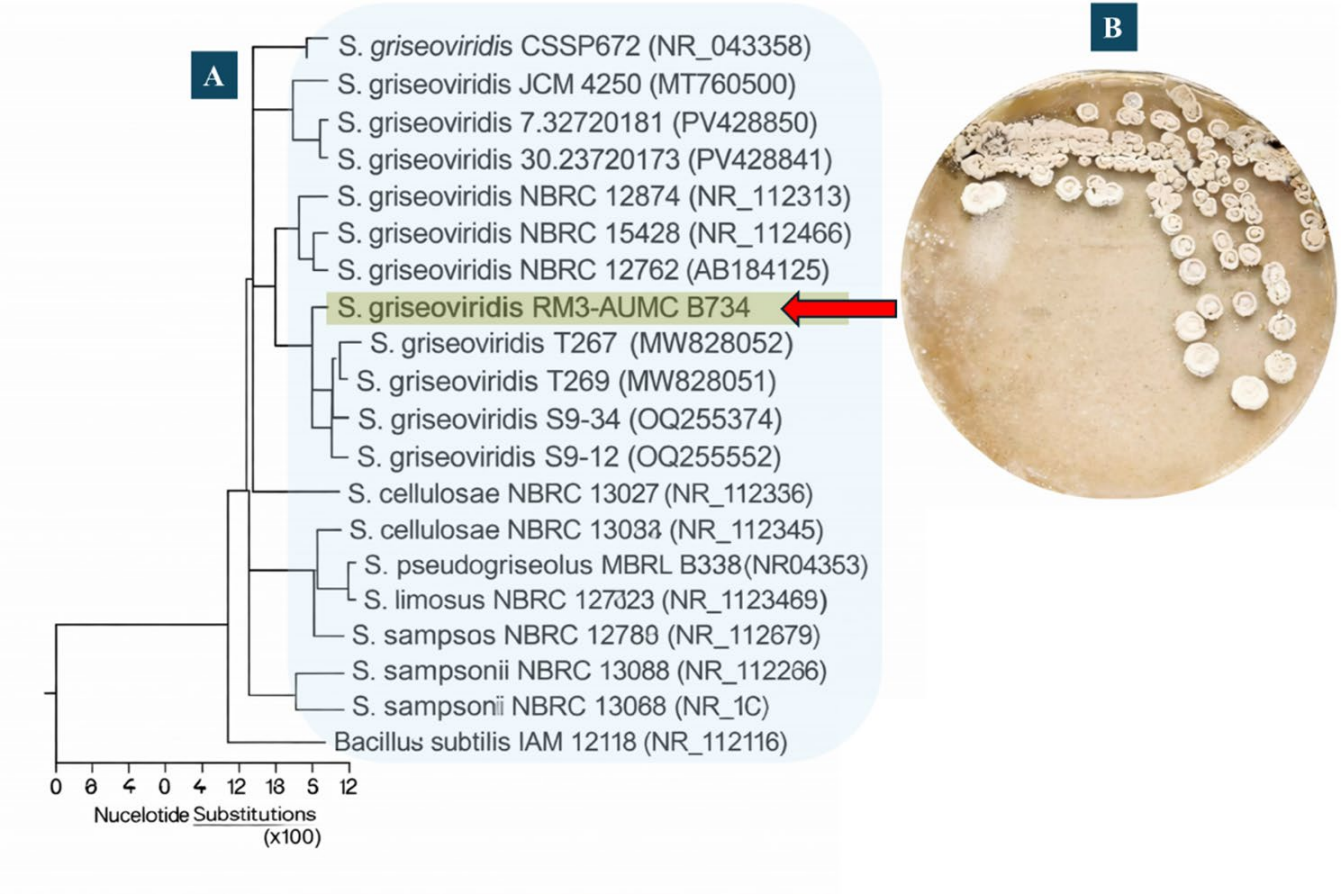
**A**) Phylogenetic tree based on 16 S rRNA gene sequences showing the position of *Streptomyces griseoviridis* isolate RM3 (strain AUMC B734; arrowed) among closely related taxa retrieved from GenBank. (**B**) Colony morphology of *S. griseoviridis* RM3 (AUMC B734) grown for 7 days

The actinobacterial strain RM3 (strain AUMC B734) was isolated successfully from a soil sample collected from a garden at Helwan University campus, Cairo, Egypt. Isolation was carried out on starch casein agar containing tetracycline and ampicillin to prevent bacterial contamination. The molecular identification of the actinobacterial isolate was performed through sequencing of the 16 S rRNA gene. The obtained sequence was compared with closely related sequences retrieved from the GenBank database using BLAST analysis. The isolate showed 98.98%–99.27% sequence identity and 98%–100% query coverage with several strains of *Streptomyces griseoviridis*, including the type strain *S. griseoviridis* NBRC 15,428 (accession number NR_112466). A phylogenetic tree (Fig. 1) was constructed to visualize the evolutionary relationships, with *Bacillus subtilis* serving as an outgroup. This analysis confirmed the placement of the isolate within the *S. griseo*viridis clade. The 16 S rRNA gene sequence of isolate RM3 has been deposited in GenBank under the accession number PX851554.

### Phytohormone composition of the CFF

HPLC analysis of the CFF confirmed the production of major phytohormones by the *Streptomyces griseoviridis* isolate, with quantified concentrations as follows: GA₃ at 46.68 µg/100 mL, zeatin at 18.90 µg/100 mL, IAA at 3.93 µg/100 mL, and ABA at 0.386 µg/100 mL (Table 1).

**Table 1:** Phytohormone concentrations in the *Streptomyces griseoviridis* filtrate

Hormones	Mean Conc (µg/100 ml) ± SE
Zeatin	18.90 ± 0.53
Gibberellin (GA3)	46.68 ± 1.20
Indole-3-acetic acid	3.93 ± 0.56
Abscisic Acid	0.38 ± 0.006

### GC-MS analysis

The CFF of *Streptomyces griseoviridis* culture filtrate was subjected to GC-MS analysis to identify the major volatile and semi-volatile organic compounds. A total of 16 major peaks were detected and identified based on their retention times (RT) and mass spectral fragmentation patterns. The most abundant compound was oleic acid (9‑octadecenoic acid (Z)-), accounting for 17.16% of the total peak area. Other predominant fatty acids and their derivatives included hexadecenoic acid methyl ester (palmitic methyl ester, 8.34%), 9‑octadecenoic acid methyl ester (oleic methyl ester, 5.90%), and 9‑hexadecenoic acid methyl ester (palmitoleic methyl ester, 1.65%). Several phenolic compounds, such as 4‑methoxy‑1,2‑benzenediol (1.70%) and 1,2,4‑benzenetriol (1.88%), were also detected. Additionally, minor amounts of long-chain fatty acid esters and a triglyceride derivative were observed (Table 2).

**Table 2:** List of identified compounds from the GC-MS analysis of the CFF of *Streptomyces griseoviridis*

Code	Compound	RT (min)	Mean Area % ±SE
ED01	4-Methoxy-1,2-benzenediol	10.63	1.70 ± 0.09
ED02	1,2,4-Benzenetriol	15.14	1.88 ± 0.12
ED03	1,4-Benzenediol derivative (2-tert-butyl-5-allyl)	16.49	1.90 ± 0.08
ED04	2-Acetyl-3-(2-cinnamido)ethyl-7-methoxyindole	20.78	0.25 ± 0.04
ED05	Hexadecanoic acid, 2,3-dihydroxypropyl ester	22.46	0.93 ± 0.06
ED06	Tetradecanoic acid (Myristic acid)	22.96	0.93 ± 0.04
ED07	9-Hexadecenoic acid, methyl ester (Palmitoleic methyl ester)	24.81	1.65 ± 0.08
ED08	Hexadecanoic acid, methyl ester (Palmitic methyl ester)	25.37	8.34 ± 0.58
ED09	9-Octadecenoic acid, methyl ester (Oleic methyl ester)	28.72	5.90 ± 0.44
ED10	Oleic acid/9-Octadecenoic acid (Z)-	30.37	17.16 ± 1.19
ED11	Hexadecadienoic acid, methyl ester	30.52	0.83 ± 0.08
ED12	cis-11-Eicosenoic acid/methyl ester	32.13	0.55 ± 0.04
ED13	Docosanoic acid, methyl ester (Behenic methyl ester)	37.19	0.44 ± 0.03
ED14	9,12,15-Octadecatrienoic acid derivatives (linolenic)	42.32	1.40 ± 0.04
ED15	9-Octadecenoic acid, 1,2,3-propanetriyl ester (triglyceride)	42.72	0.24 ± 0.03
ED16	16-Octadecenoic acid, methyl ester	44.93	0.37 ± 0.06

### Effects of seed priming with *Streptomyces griseoviridis* culture filtrate on germination, growth, photosynthetic pigments, and soluble metabolites in *Phaseolus vulgaris* and *Vicia faba*

**Fig. 2:**
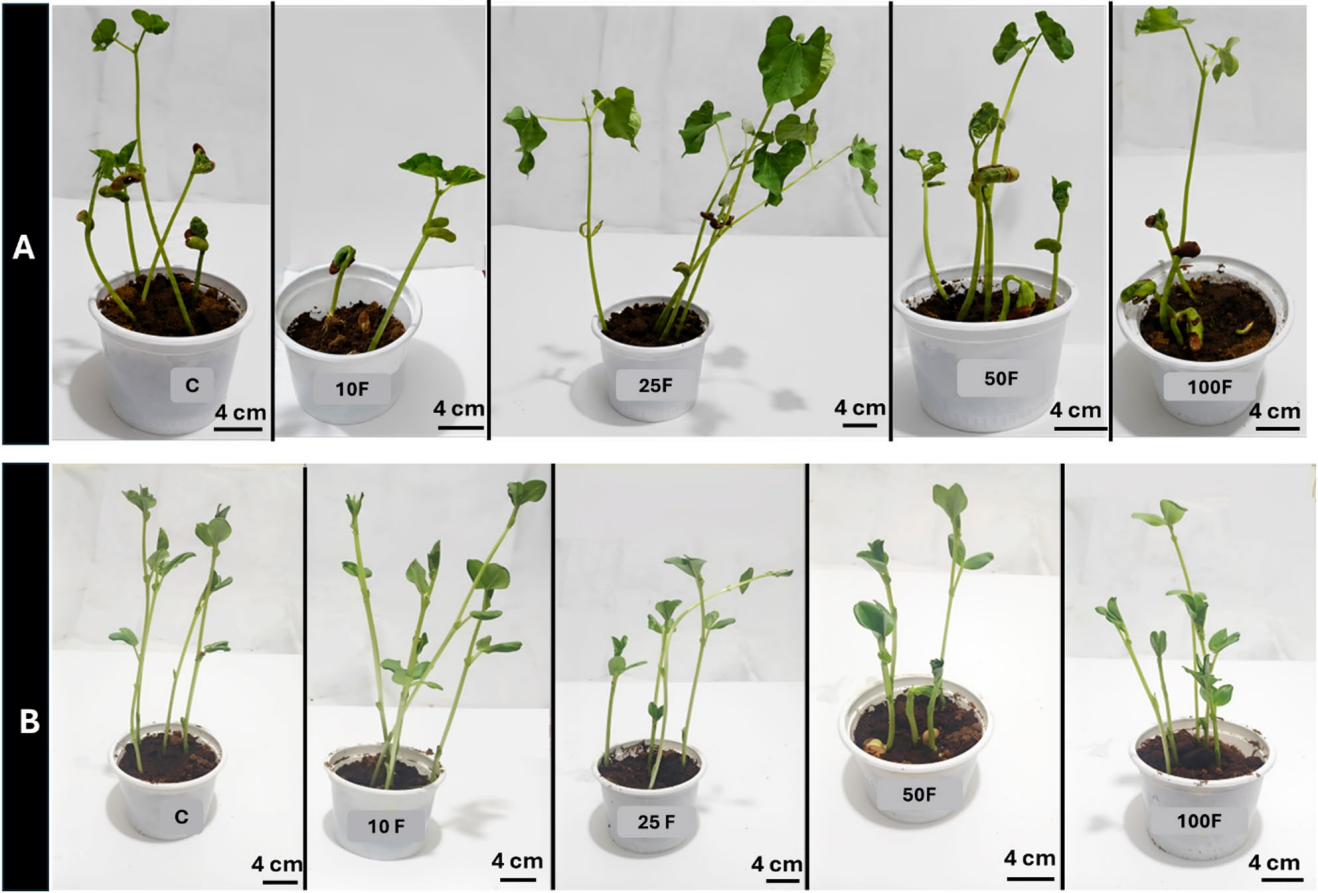
Seedlings of *Phaseolus vulgaris* (**A**) and *Vicia faba* (**B**) grown under different concentrations of *Streptomyces griseoviridis* culture filtrate. Treatments include C (control; 0% filtrate), 10 F (10% filtrate), 25 F (25% filtrate), 50 F (50% filtrate), and 100 F (100% filtrate)

Seed priming with *S. griseoviridis* CFF showed significant, species-specific and concentration-dependent effects on germination indices and growth (Fig. 2). In *Phaseolus vulgaris*, treatment with 25% actinobacterial culture filtrate resulted in a noticeable improvement in germination performance compared with the untreated control. Specifically, germination percentage increased to 66.7 ± 3.33%, whereas the control exhibited 60.0 ± 2.89%.

**Fig. 3:**
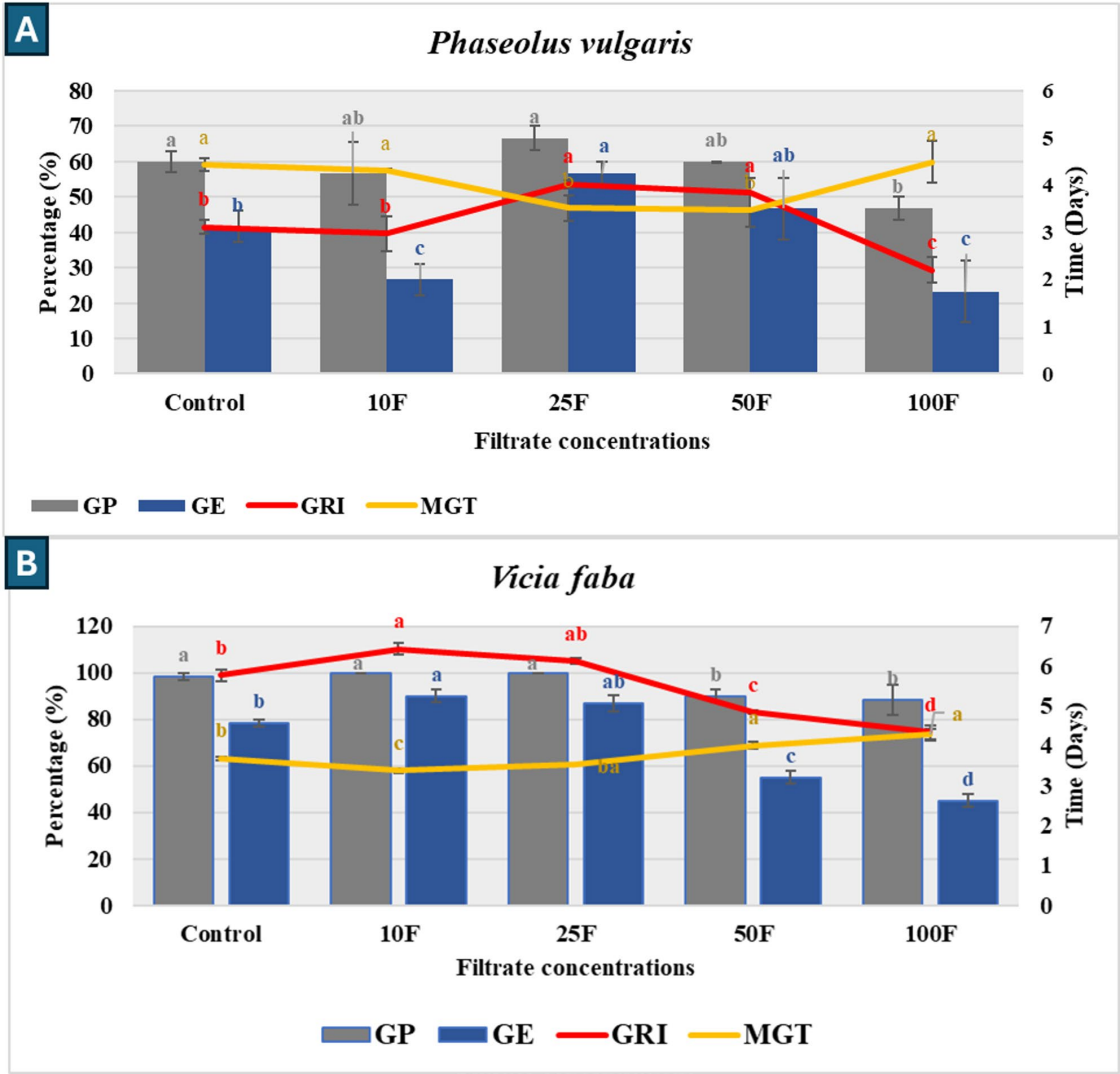
Effect of increasing *Streptomyces griseoviridis* filtrate concentration on key germination parameters of *Phaseolus vulgaris* (**A**) and *Vicia faba* (**B**). Treatments included control (0% filtrate), 10F (10% filtrate), 25F (25% filtrate), 50F (50% filtrate), and 100F (100% filtrate). The x-axis represents filtrate concentration, while the left y-axis indicates percentage values for Germination Percentage (GP), Germination Energy (GE), and Germination Rate Index (GRI), and the right y-axis represents time (days) for Mean Germination Time (MGT). Error bars represent the standard error of the mean. Different letters above the bars and lines indicate statistically significant differences among treatments (*p* < 0.05), as determined by one-way analysis of variance (ANOVA) followed by Tukey’s Honest Significant Difference (HSD) post hoc test

Similarly, the 25% filtrate concentration significantly enhanced germination energy (GE) and germination rate index (GRI), reaching 56.7 ± 3.33 and 4.02 ± 0.08, respectively, compared with 41.7 ± 4.41 and 3.11 ± 0.15 in the control treatment. Mean germination time (MGT) was not significantly affected by the 25% filtrate application (Fig. 3A).

In *Vicia faba*, seed priming with 10% and 25% culture filtrate resulted in 100% germination, compared with 98.3 ± 1.67% in the control treatment. In contrast, higher concentrations (50% and 100%) significantly reduced germination percentage to 95% (Fig. 3B). Furthermore, the 10% CFF markedly enhanced germination vigor, as reflected by a significant increase in GE and GRI, which reached 90.0 ± 2.89 and 6.43 ± 0.15, respectively. This treatment also significantly reduced MGT to 3.38 ± 0.06 days, indicating an accelerated germination process compared with the control.

**Fig. 4:**
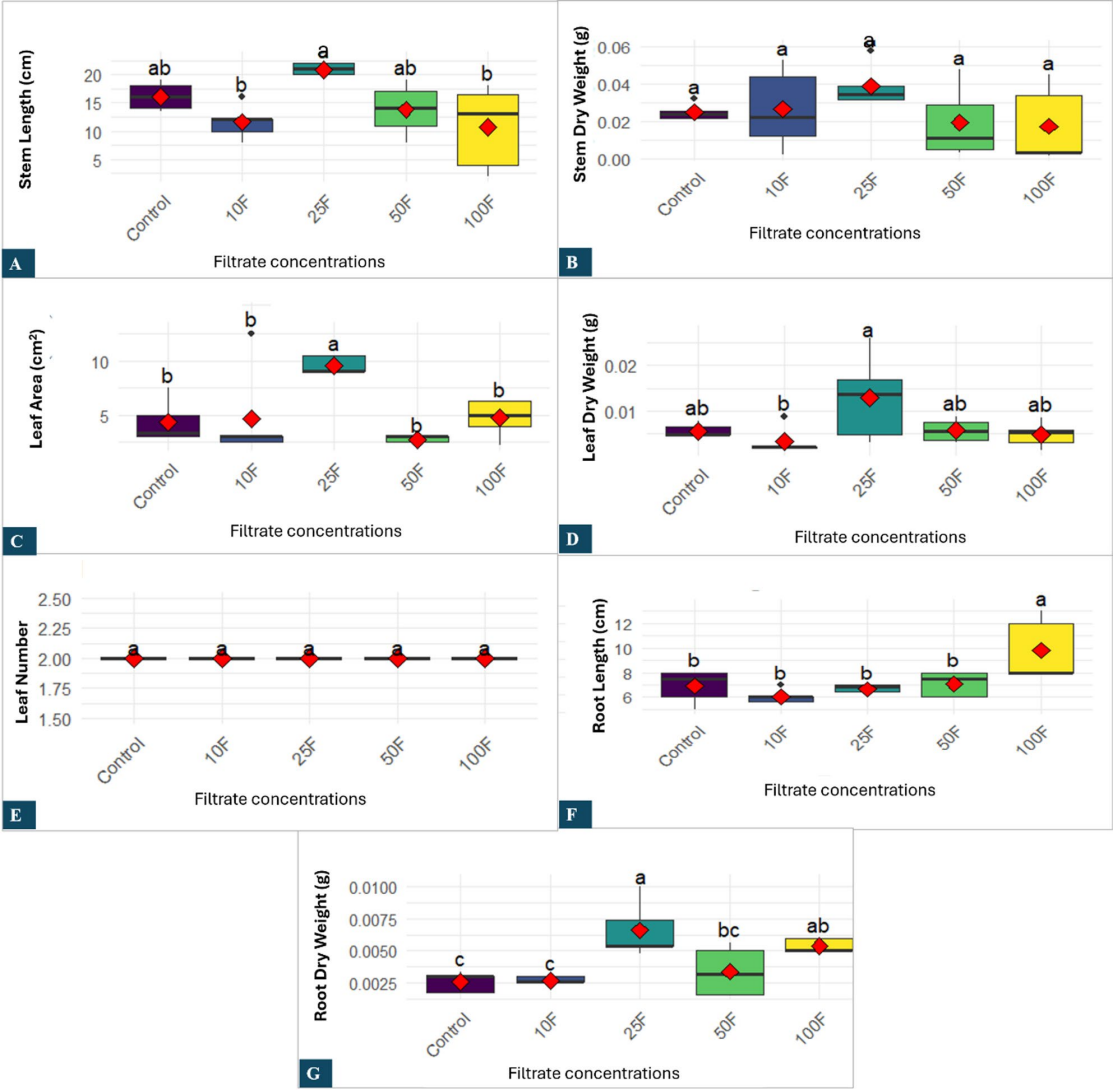
Effect of increasing *Streptomyces griseoviridis* filtrate concentration on growth parameters of *Phaseolus vulgaris*. Treatments included control, 10F (10% filtrate), 25F (25% filtrate), 50F (50% filtrate), and 100F (100% filtrate). **A** Stem length; **B** stem dry weight; **C** leaf area; **D** leaf dry weight; **E** leaf number; **F** root length; and **G** root dry weight. The central diamond indicates the mean value for each treatment group. Different letters above the bars denote statistically significant differences between treatments (*p* < 0.05), as determined by one-way ANOVA followed by Tukey’s Honest Significant Difference (HSD) post-hoc test

In *Phaseolus vulgaris* (Fig. 4), seed priming with 25% CFF markedly enhanced biomass accumulation and vegetative growth parameters compared with the control. Stem length increased significantly to 20.80 ± 0.58 cm, relative to 16.10 ± 0.98 cm in the untreated plants. This treatment also resulted in a significant increase in leaf area (9.55 ± 0.37 cm²) and leaf dry weight (0.01288 ± 0.00423 g) compared with the control. Regarding root traits, the highest concentration (100% CCF) significantly increased root length, whereas the 25% CCF treatment was more effective in enhancing root dry weight.

**Fig. 5:**
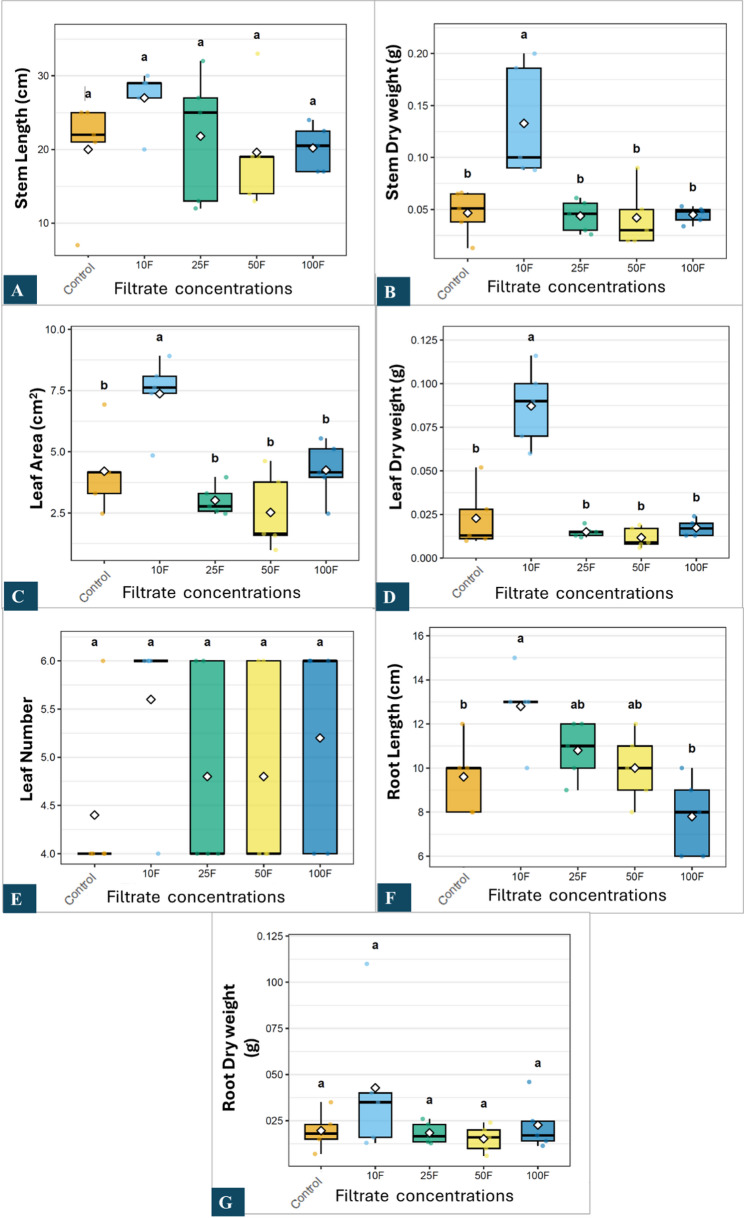
Effect of increasing *Streptomyces griseoviridis* filtrate concentrations on growth parameters of *Vicia faba*. Treatments included control, 10F (10% filtrate), 25F (25% filtrate), 50F (50% filtrate), and 100F (100% filtrate). **A** Stem length; **B** stem dry weight; **C** leaf area; **D** leaf dry weight; **E** leaf number; **F** root length; and **G** root dry weight. Data points represent individual measurements, with the central diamond indicating the mean value for each treatment group. Different letters above the bars denote statistically significant differences between treatments (*p* < 0.05), as determined by one-way ANOVA followed by Tukey’s Honest Significant Difference (HSD) post-hoc test

*In Vicia faba*, seed priming with the 10% CFF significantly enhanced multiple morphological parameters (Fig. 5). Although stem length was highest in the 10% CFF treatment (27.00 ± 4.06 cm), followed by 25% CFF (21.80 ± 8.87 cm) and the control (21.60 ± 8.76 cm), these differences were not statistically significant (Fig. 5). In contrast, stem biomass was strongly influenced by treatment; 10% CFF produced a significantly higher stem dry weight (0.1328 ± 0.0554 g) compared with all other concentrations. Leaf development was similarly enhanced by 10 F, which yielded the largest leaf area (7.372 ± 1.524 cm²) and the highest leaf dry weight (0.0872 ± 0.0226 g), whereas values for higher concentrations (25, 50, 100% CFF) and the control were statistically similar. Root growth also responded positively to 10% CFF priming. Root length was maximal at 12.80 ± 1.79 cm, significantly exceeding the control (9.60 ± 1.67 cm) and 100% CFF (7.80 ± 1.79 cm), while intermediate concentrations (25% CFF: 10.80 ± 1.30 cm; 50% CFF: 10.00 ± 1.58 cm) showed non-significant differences relative to 10% CFF and the control, indicating a dose-dependent effect. Similarly, root dry weight was highest under 10% CFF treatment (0.0556 ± 0.0309 g), significantly surpassing all other treatments, which did not differ statistically from one another. Overall, these results demonstrate that a moderate 10% CFF concentration optimally enhances both shoot and root growth in *V. faba*, whereas higher concentrations confer no additional benefit.

Analysis of photosynthetic pigments further elucidated species-specific response patterns. In *Phaseolus vulgaris* (Table 3), pigment accumulation increased at the 25% CFF, with chlorophyll a increasing to 1.073 ± 0.085 mg/g, chlorophyll b to 0.495 ± 0.040 mg/g, and carotenoids to 2.234 ± 0.160 mg/g, yielding a total pigment content of 3.792 ± 0.285 mg/g, substantially higher than the control value of 2.816 ± 0.141 mg/g. The carotenoid-to-chlorophyll ratio also reached a maximum of 1.434 ± 0.075 under the 25% treatment, compared to 1.130 ± 0.045 in the control. Lower concentrations, such as 10%, resulted in reduced pigment levels (total pigments: 2.289 ± 0.140 mg/g), while the 100% treatment caused a notable decrease to 2.408 ± 0.155 mg/g.

**Table 3:** Effects of *Streptomyces griseoviridis* culture free filtrate concentration on photosynthetic pigment content in *Phaseolus vulgaris* leaves

Treat	Chlorophyll a (mg/g)	Chlorophyll b (mg/g)	Chlorophyll a+ Chlorophyll b (mg/g)	Carotenoids (mg/g)	Total Pigment (mg/g)	Carotenoids/Chlorophyll a+ Chlorophyll b
C	0.903 ± 0.045^a^	0.419 ± 0.021^ab^	1.322 ± 0.066^ab^	1.494 ± 0.075^b^	2.816 ± 0.141^bc^	1.130 ± 0.045^b^
10 F	0.826 ± 0.050^a^	0.341 ± 0.022^b^	1.167 ± 0.070^b^	1.122 ± 0.073^b^	2.289 ± 0.140^c^	0.962 ± 0.050^c^
25 F	1.073 ± 0.085^a^	0.495 ± 0.040^a^	1.558 ± 0.120^a^	2.234 ± 0.160^a^	3.792 ± 0.285^a^	1.434 ± 0.075^a^
50 F	1.061 ± 0.075^a^	0.470 ± 0.035^ab^	1.511 ± 0.105^ab^	2.068 ± 0.145^a^	3.579 ± 0.250^ab^	1.369 ± 0.065^a^
100 F	0.814 ± 0.055^a^	0.356 ± 0.025^b^	1.170 ± 0.078^b^	1.238 ± 0.080^b^	2.408 ± 0.155^c^	1.058 ± 0.050^bc^

Treatments included C (control), 10 F (10% filtrate), 25 F (25% filtrate), 50 F (50% filtrate), and 100 F (100% filtrate)

Different letters denote statistically significant differences between treatments (*p* < 0.05), as determined by one-way ANOVA followed by Tukey’s Honest Significant Difference (HSD) post-hoc test

In *Vicia faba*, the 10% CFF treatment significantly increased chlorophyll a content from 0.689 ± 0.032 mg/g in the control to 0.992 ± 0.065 mg/g, chlorophyll b from 0.295 ± 0.015 mg/g to 0.389 ± 0.028 mg/g, and carotenoids from 0.886 ± 0.041 mg/g to 1.729 ± 0.110 mg/g. This resulted in a total pigment content of 3.110 ± 0.195 mg/g under the 10% treatment, compared to 1.869 ± 0.083 mg/g in the control. The carotenoid-to-total chlorophyll ratio also significantly increased from 0.901 ± 0.035 to 1.253 ± 0.045. The 25% treatment maintained elevated pigment levels, with total pigments at 2.944 ± 0.245 mg/g, whereas higher concentrations led to a decline; the 50% and 100% treatments reduced total pigments to 2.249 ± 0.160 mg/g and 1.781 ± 0.097 mg/g, respectively (Table 4).

**Table 4:** Effects of *Streptomyces griseoviridis* culture filtrate concentration on photosynthetic pigment content in *Vicia faba* leaves

Treat	Chlorophyll a (mg/g)	Chlorophyll b (mg/g)	Chlorophyll a+ Chlorophyll b (mg/g)	Carotenoids (mg/g)	Total Pigment (mg/g)	Carotenoids/Chlorophyll a+ Chlorophyll b
C	0.689 ± 0.032^b^	0.295 ± 0.015^ab^	0.983 ± 0.045^bc^	0.886 ± 0.041^b^	1.869 ± 0.083^c^	0.901 ± 0.035^b^
10 F	0.992 ± 0.065^a^	0.389 ± 0.028^a^	1.381 ± 0.090^a^	1.729 ± 0.110^a^	3.110 ± 0.195^a^	1.253 ± 0.045^a^
25 F	0.972 ± 0.085^a^	0.360 ± 0.035^ab^	1.332 ± 0.115^ab^	1.612 ± 0.135^a^	2.944 ± 0.245^ab^	1.211 ± 0.055^a^
50 F	0.809 ± 0.055^ab^	0.311 ± 0.025^ab^	1.121 ± 0.078^abc^	1.128 ± 0.085^b^	2.249 ± 0.160^bc^	1.006 ± 0.040^b^
100 F	0.691 ± 0.035^b^	0.260 ± 0.020^b^	0.951 ± 0.052^c^	0.830 ± 0.048^b^	1.781 ± 0.097^c^	0.873 ± 0.030^b^

Treatments included C (control), 10 F (10% filtrate), 25 F (25% filtrate), 50 F (50% filtrate), and 100 F (100% filtrate)

Different letters denote statistically significant differences between treatments (*p* < 0.05), as determined by one-way ANOVA followed by Tukey’s Honest Significant Difference (HSD) post-hoc test

The CFF also significantly influenced soluble protein and sugar content in a species- and concentration-specific manner (Table 5). In *Phaseolus vulgaris*, the 25 F and 50 F treatments produced the highest soluble protein concentration at 165.4 ± 4.13 and 136.84 ± 2.8 mg/g dry weight, significantly exceeding the control value of 108.88 ± 6.57 mg/g dry weight. The 10% and 100% CFF did not differ significantly from the control. Soluble sugars increased *P. vulgaris* with the 100% treatment reaching 57.96 ± 6.9 mg/g, significantly higher than the control. For *Vicia faba*, soluble protein was maximized under the 10% treatment, with values of 295.37 ± 33.6 mg/g, which was significantly greater than the control (202.11 ± 14.13 mg/g). Soluble sugar content in *V. faba* was highest under the 100% treatment at 66.69 ± 4.06 mg/g, significantly above the control (42.5 ± 4.25 mg/g).

**Table 5:** Mean soluble protein concentration and soluble sugars in leaf tissues of *P. vulgaris* and *V. faba* treated with varying concentrations of *Streptomyces griseoviridis* filtrate

Treat	Soluble protein (mg/g dry weight)		Soluble Sugars (mg/g dry weight)	
	*Phaseolus* *vulgaris*	*Vicia* *faba*	*Phaseolus vulgaris*	*Vicia* *faba*
C	108.88 ± 6.57^c^	202.11 ± 14.3^b^	30.8 ± 5.02^b^	42.5 ± 4.25^c^
10 F	109.02 ± 3.02^c^	295.37 ± 33.68^a^	44.02 ± 16.10^ab^	52.99 ± 2.95^b^
25 F	165.42 ± 4.13^a^	229.88 ± 71.66^ab^	53.34 ± 3.43^ab^	46.99 ± 0.085^bc^
50 F	136.84 ± 2.80^b^	263.76 ± 28.52^ab^	50.65 ± 4.66^ab^	53.17 ± 6.74^b^
100 F	107.88 ± 3.20^c^	212.32 ± 20.72^b^	57.96 ± 6.91^a^	66.69 ± 4.06^a^

Treatments included C (control), 10 F (10% filtrate), 25 F (25% filtrate), 50 F (50% filtrate), and 100 F (100% filtrate)

Different letters denote statistically significant differences between treatments (*p* < 0.05), as determined by one-way ANOVA followed by Tukey’s Honest Significant Difference (HSD) post-hoc test

### Molecular docking analysis

**Fig. 6:**
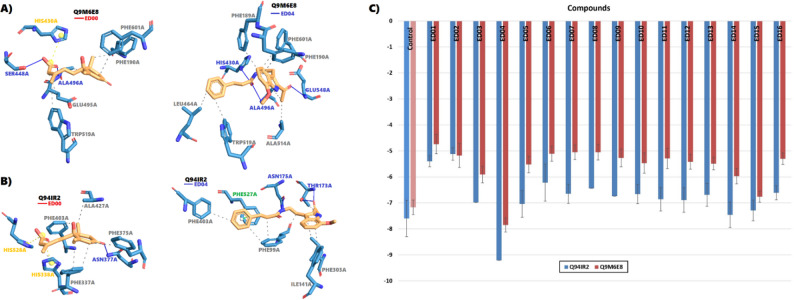
PLIP-based interaction analysis and docking affinity comparison for carotenoid oxygenase targets. (**A**) Interaction profile of control ligand ED00 and ED04 within Q9M6E8 (NCED1) (**B**) Interaction network of ED00 and ED04 within Q94IR2 (CCD1) (**C**) Comparative binding affinity (kcal/mol) of ED compounds against Q9M6E8 and Q94IR2

**Fig. 7:**
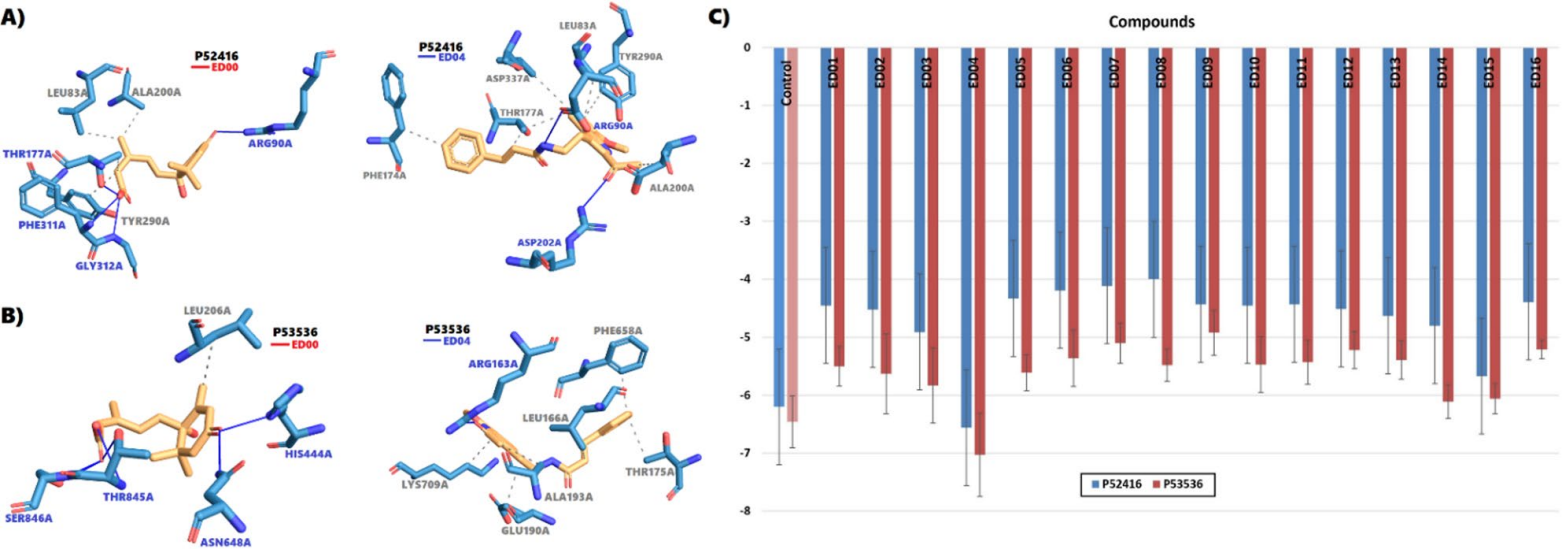
Docking and PLIP interaction analysis for starch metabolism targets. (**A**) Control ligand ED00 and ED04 interactions in P52416 (AGPase) (**B**) ED00 and ED04 interaction profile in P53536 (PHO1) (**C**) Comparative binding affinities of ED compounds for P53536 and P52416

Molecular docking against four plant protein targets—NCED1 (Q9M6E8), CCD1 (Q94IR2), plastidial starch phosphorylase (P53536), and ADP‑glucose pyrophosphorylase small subunit (P52416)—revealed that 2‑Acetyl‑3‑(2‑cinnamido)ethyl‑7‑methoxyindole (ED04) consistently exhibited the strongest binding affinity among all tested ligands (Fig. 6C). For NCED1, this compound achieved a mean binding energy of − 7.85 ± 0.27 kcal/mol, compared to − 7.17 ± 0.28 kcal/mol for the control ligand; for CCD1, it gave − 9.21 ± 1.27 kcal/mol versus − 7.60 ± 0.70 kcal/mol for the control. Most fatty acid derivatives showed much weaker binding (‑4.74 to − 6.76 kcal/mol), with only 9,12,15‑octadecatrienoic acid derivatives (linolenic acid) and 9‑Octadecenoic acid, 1,2,3‑propanetriyl ester (triglyceride) (ED14 and ED15) displaying moderate improvement. Protein‑ligand interaction profiling (PLIP) showed that in NCED1, 2‑Acetyl‑3‑(2‑cinnamido)ethyl‑7‑methoxyindole formed hydrophobic contacts with seven residues (Phe189, Phe190, Leu464, Ala496, Ala514, Trp519, Phe601) and hydrogen bonds with His430, Ala496, and Glu548, whereas the control ligand relied on a smaller polar network including a salt bridge to His430 (Fig. 6A). In CCD1, the same compound engaged six hydrophobic residues (Phe99, Ile141, Thr173, Phe303, Phe403, and Phe527), two strong hydrogen bonds with Thr173 (donor–acceptor distances 2.82 Å and 3.14 Å, angles > 145°); and a parallel π‑stacking interaction with Phe527 (centroid distance 3.94 Å, angle 13.25°). The control ligand formed only a single hydrogen bond and two salt bridges (Fig. 6B). For the starch metabolism targets, 2‑Acetyl‑3‑(2‑cinnamido)ethyl‑7‑methoxyindole bound to P53536 with an affinity of − 7.03 ± 1.33 kcal/mol (control: − 6.46 ± 0.62) and to P52416 with − 6.56 ± 0.72 kcal/mol (control: − 6.20 ± 0.45) (Fig. 7C). In P52416, it expanded the hydrophobic contact network from four to seven residues while retaining a strong hydrogen bond with Arg90 (Fig. 7A); in P53536, it shifted from six hydrogen bonds (control) to a single hydrogen bond with Arg163 and six hydrophobic contacts, including Phe658 (Fig. 7B). All interactions met strict geometric criteria for validity (hydrogen bonds: donor–acceptor < 3.5 Å, angle > 120°; π‑stacking: centroid distance < 4.5 Å, angle < 30°).

## Discussion

Plant growth–promoting actinobacteria represent an important group of rhizosphere-associated microorganisms capable of modulating plant development through the production of phytohormones and bioactive metabolites. In the present study, the culture filtrate fraction of *Streptomyces griseoviridis* demonstrated pronounced, species-specific, and concentration-dependent effects on seed germination, vegetative growth, pigment accumulation, and primary metabolism in *Phaseolus vulgaris* and *Vicia faba*.

The successful isolation and molecular identification of *Streptomyces griseoviridis* from garden soil in Cairo confirms the ecological adaptability and cosmopolitan distribution of plant-associated actinobacteria in terrestrial ecosystems. Members of the genus *Streptomyces* are frequently isolated from agricultural and rhizosphere soils due to their ability to form resistant spores and to compete effectively under a variety of environmental conditions. Recent studies have highlighted the importance of isolating native *Streptomyces* strains as a source of plant growth-promoting and stress-tolerant microorganisms for sustainable agriculture [29]. Kadirova et al. [30]demonstrated that soil-derived Streptomyces isolates from saline environments possess considerable physiological diversity and adaptation potential, emphasising the value of local isolation programs for the selection of agriculturally beneficial strains. Similarly, Ye et al. [31] reported that the newly isolated Streptomyces strains showed multiple plant growth-promoting traits, including the production of bioactive metabolites and enhancement of plant development. Phylogenetic placement of isolate RM3 within the *S. griseoviridis* clade further supports its potential as a biologically active strain with applications in plant growth promotion and biostimulant development. The growing interest in microbial phytohormone-producing microorganisms has also reinforced the importance of strain-level characterization, as closely related *Streptomyces* isolates may vary greatly in their metabolic capabilities and plant-associated functions [29]. In the present study, 16 S rRNA gene sequencing enabled a reliable taxonomic assignment of isolate RM3 within the *S. griseoviridis* clade in accordance with current polyphasic approaches applied for the characterization of novel Streptomycetes [32]. In addition, there is growing evidence that populations of plant-associated Streptomyces constitute important members of the rhizosphere microbiome involved in plant growth promotion, disease suppression and adaptation to environmental stress.

High-performance liquid chromatography analysis confirmed the presence of major phytohormones in the CFF, including cytokinins, auxins, gibberellins, and abscisic acid. While IAA production has been reported in several *Streptomyces* species [33] as well as in other actinobacterial taxa [34], and the biosynthesis of gibberellins, abscisic acid (ABA), and cytokinins has been broadly documented among actinobacteria [35], comprehensive qualitative and quantitative in vitro phytohormone data specific to *S. griseoviridis* are notably limited. It is of particular interest that a prior investigation into *S. griseoviridis* isolated from the bean rhizosphere reported an inability to detect common plant growth regulators, including IAA, indole-pyruvic acid, GA, and zeatin, in the culture filtrates of tested strains under in vitro conditions [36]. Microbial seed priming is an emerging sustainable agricultural strategy that leverages beneficial microorganisms to improve seed germination, early seedling establishment, and vigor, largely through the delivery of phytoactive metabolites and bioactive regulatory compounds [4]. In this study, seed priming with a CFF derived from *Streptomyces griseoviridis* resulted in significant, species-specific, and concentration-dependent enhancements in early seedling growth of the two legume crops *Phaseolus vulgaris* and *Vicia faba*.

Our data indicate that a 25% concentration of the actinobacterial filtrate significantly enhanced all measured germination indices in *Phaseolus vulgaris*, including germination percentage, germination rate index, and mean germination time, whereas a 10% concentration of the bacterial filtrate improved the germination of *Vicia faba*. This species-specific and concentration-dependent effect can be attributed to the phytohormones present in the *S. griseoviridis* filtrate. Cytokinin and gibberellin are among the principal phytohormones governing seed germination. Gibberellic acids trigger the hydrolysis of stored seed reserves by inducing hydrolytic enzymes such as α-amylase while also mediating the degradation of DELLA proteins, key repressors of germination [5, 6]. Cytokinins complement this process by promoting cell division and enhancing GA-mediated signaling pathways [37, 38]. At a 100% concentration of the actinobacterial filtrate, germination parameters declined in both plant species, an effect attributed to the presence of abscisic acid. As a principal inhibitor of seed germination [39], ABA suppresses germination-promoting pathways at elevated concentrations, overriding the stimulatory effects of other phytohormones present in the filtrate. The antagonistic balance of ABA and gibberellins (GAs) serves as a central hub for transducing environmental cues into the decision to maintain dormancy or initiate germination [40]. This indicates a concentration-dependent, hormetic response where the balance between stimulatory and inhibitory signals determines germination outcomes.

Microbial filtrate priming significantly improved shoot morphogenesis, biomass accumulation, leaf expansion, and pigment biosynthesis in the tested legumes, but the magnitude of response differed between species. *Phaseolus vulgaris* exhibited its strongest stimulation at 25% filtrate, while *Vicia faba* achieved optimal enhancement at 10% filtrate, demonstrating a clear species-dependent dose sensitivity. The superior response of *P. vulgaris* to 25% filtrate may indicate a higher tolerance or requirement for microbial hormone enrichment, whereas *V. faba* appears more responsive to moderate filtrate exposure, consistent with reports that cytokinin-driven growth regulation follows species-specific, non-linear dose–response curves governed by endogenous hormone homeostasis and receptor sensitivity [34, 40]. Although *Streptomyces* spp. are generally recognized as effective root colonizers, this capacity is not universal, and colonization efficiency can vary considerably among species. Therefore, a critical prerequisite for selecting any *Streptomyces* strain as a plant growth-promoting agent is to first assess its ability to enhance plant growth under relevant environmental conditions [1].

Numerous plant-associated bacteria are capable of producing modulatory phytohormones, including cytokinins, auxins, gibberellins, and abscisic acid, which serve as signaling molecules that can reprogram host metabolic pathways. For example, phytohormone-producing rhizobacteria are well known to synthesize auxins, cytokinins, gibberellins, and even ABA, thereby influencing a range of growth and stress responses in their hosts [41]. A key mechanistic explanation for the observed phenotypes is the presence of zeatin in *Streptomyces griseoviridis*. Zeatin acts as a potent developmental signal in plants, stimulating mitotic reactivation in shoot apical and axillary meristems, enhancing shoot growth and lateral bud activity, and influencing chloroplast maintenance and leaf expansion through modulation of growth processes, while its root-to-shoot translocation in the xylem contributes to the regulation of leaf size and overall shoot morphology [42].

The CFF significantly enhanced soluble protein and sugar metabolism in both legumes. Cytokinins and IAA produced by *Streptomyces* spp. have been shown to stimulate chloroplast biogenesis and nutrient uptake and improve photosynthetic activity, indirectly improving nitrogen fixation and protein synthesis through increased cellular energy and carbon skeleton availability [1, 43]. Also, zeatin has been reported to improve crude protein in sorghum under stress conditions [2]. Furthermore, *Streptomyces*-derived metabolites such as organic acids and antioxidative molecules have been reported to influence the metabolic profiling of treated plants [43, 44].

In addition to its known capacity for hormone production, the growth-promoting activity of *Streptomyces griseoviridis* can be attributed to a diverse repertoire of bioactive metabolites identified through GC-MS analysis of its filtrate extract. These compounds, which include phenolic molecules, free fatty acids, and fatty acid esters, provide a compelling chemical basis for the observed enhancement of legume germination and seedling vigor. Among these, oleic acid and palmitic acid emerged as the most abundant constituents, representing 17.16% and 8.34% of the detected metabolome (as methyl esters), respectively. The role of these fatty acids in promoting early plant development is well-supported by experimental evidence. In pot trials involving cucumber and tomato, soil amended with a combination of oleic and palmitic acids significantly improved seedling performance, resulting in greater stem diameter, increased biomass, enhanced lateral root formation, and higher root vigor compared to untreated controls. Furthermore, the application of oleic acid has been shown to mitigate the phytotoxic effects of total petroleum hydrocarbon contamination in soil, improving soil quality and promoting rice germination under stress conditions [45].

Furthermore, linolenic acid warrants attention as growth-promoting metabolite. It is critically required as the precursor for jasmonate signaling, which regulates the final maturation and release of pollen, making it critical for plant fertility and seed production [46]. Furthermore, it plays a role in inducing secondary laticifer differentiation in *Hevea brasiliensis* [47].

Moreover, the phenolic compounds 4-methoxy-1,2-benzenediol and 1,2,4-benzenetriol have been identified in the culture filtrate. Phenolic compounds have been extensively documented for their ability to enhance plant growth and improve crop productivity. Evidence from numerous studies indicates that these metabolites exert growth-promoting effects by positively influencing multiple physiological and developmental processes, including seed germination, shoot and root elongation, biomass accumulation, photosynthetic pigment content, and overall plant metabolism [48, 49].

The concentration of phenolic compounds has also been shown to critically influence their biological activity, with low concentrations typically stimulating plant growth while higher concentrations exert inhibitory effects. Several mechanisms have been proposed to explain their growth-promoting activity, including promoting cell wall formation by serving as lignin precursors or stimulating lignin biosynthesis, regulating the synthesis and degradation of auxin in plant tissues, stimulating leaf expansion, and promoting callus proliferation and enhancing root growth [50].

The docking study was performed to evaluate the binding interactions of the CFF constituents—particularly 2-Acetyl-3-(2-cinnamido)ethyl-7-methoxyindole—with four plant protein targets (NCED1, CCD1, P53536, P52416) mechanistically linked to seed germination and pigmentation. The superior binding of this indole derivative across both carotenoid oxygenases and starch-metabolism enzymes can be explained by its structural features—aromatic conjugation, conformational rigidity from the indole scaffold, and balanced hydrogen-bond donor/acceptor capacity—which enable favorable complementarity with extended catalytic grooves or substrate-access tunnels. For NCED1, the expanded hydrophobic network and additional polar contacts of 2-Acetyl-3-(2-cinnamido)ethyl-7-methoxyindole compensate for the loss of the Ser448 interaction seen with the control, suggesting a tunnel-filling mode that would reduce endogenous ABA levels by inhibiting the committed step of ABA biosynthesis, thereby weakening the hormonal brake on germination [51–53]. In CCD1, the two strong hydrogen bonds with Thr173 and the parallel π-stack with Phe527, together with engagement of multiple phenylalanine residues (Phe99, Phe303, Phe403, and Phe527), indicate occlusion of the substrate-access tunnel; because CCD1 cleaves carotenoids to produce apocarotenoids involved in pigment homeostasis, such inhibition would perturb pigmentation phenotypes as observed in the bioassay [54, 55]. The absence of salt bridges to the catalytic histidines in this binding mode, yet superior binding affinity, illustrates that distributed complementarity (π-stacking, hydrophobic packing, and geometrically optimal hydrogen bonds) can outweigh isolated electrostatic anchors [27, 28]. For the starch metabolism targets, 2-acetyl-3- (2-cinnamido) ethyl-7-methoxyindole outperformed most fatty acid derivatives (e.g., hexadecanoic acid methyl ester, oleic acid methyl ester, and linolenic acid derivatives), which lacked sufficient shape complementarity despite their lipophilicity. In AGPase (P52416), the expanded hydrophobic network and maintained Arg90 hydrogen bond suggest interference with ADP-glucose substrate positioning within the nucleotidyl-transferase cleft [56, 57]. In starch phosphorylase (P53536), the dramatic shift from multiple polar anchors to a single hydrogen bond plus extensive hydrophobic contacts—including Phe658—implies that the indole compound may bind outside the PLP-binding site, potentially blocking glucan-chain access or conformational transitions required for catalysis [58, 59]. The weak performance of flexible fatty acid chains (e.g., palmitic methyl ester, oleic acid, linolenic acid) underscores that rigidity and polar functionality are critical for productive engagement with these enzymes [20, 21]. Collectively, these computational findings provide a mechanistic rationale for the germination and pigmentation changes induced by *S. griseoviridis* metabolites and identify 2-Acetyl-3-(2-cinnamido)ethyl-7-methoxyindole as a promising lead for further structure-activity studies.

## Conclusion

The culture filtrate of *Streptomyces griseoviridis* functions as a potent priming agent capable of improving legume seed germination, growth and physiological parameters in a species-specific and dose-dependent manner. The isolate demonstrated in vitro synthesis of phytohormones, supporting the regulatory potential of its filtrate. Culture-free filtrate from the isolate priming enhanced seedling vigor, biomass, and morphogenesis at low and moderate concentrations, while concentrated filtrate exposure upregulated total soluble sugars. The findings highlight the importance of strain-level hormone screening and metabolic evaluation when developing *Streptomyces*-based biostimulant strategies. These results expand current knowledge on the metabolic influence of *S. griseoviridis* and underscore that its filtrate bioactivity likely arises from integrated metabolite signaling networks, where hormonal and non-hormonal metabolites collectively influence the nitrogen–carbon interface and photosynthetic machinery. Future work should focus on transcriptional and metabolomic dissection of these regulatory effects to better define the mechanisms governing host sensitivity and metabolic outcomes.

## Data Availability

All data generated or analysed during this study are included in this published article and supplementary file. The ITS gene sequence generated in this study has been deposited in the GenBank database under accession number PX851554.

## Abbreviations

ABA: Abscisic acid
AGPase: ADP-glucose pyrophosphorylase
ANOVA: Analysis of variance
AUMC: Assuit University Mycological Center
BLAST: Basic Local Alignment Search Tool
BSA: Bovine serum albumin
BSTFA: N, O-bis(trimethylsilyl) trifluoroacetamide
CCD1: Carotenoid cleavage dioxygenase 1
CFF: Cell-free culture filtrate
DNA: Deoxyribonucleic acid
DRC: Desert Research Center
EI: Electron ionization
GA₃: Gibberellic acid
GC-MS: Gas chromatography-mass spectrometry
GE: Germination energy
GP: Germination percentage
GRI: Germination rate index
HPLC: High-performance liquid chromatography
HSD: Honestly significant difference
IAA: Indole-3-acetic acid
MGT: Mean germination time
NCBI: National Center for Biotechnology Information
NCED1: 9-cis-epoxycarotenoid dioxygenase 1
OD: Optical density
PCR: Polymerase chain reaction
PGP: Plant growth-promoting
PLIP: Protein-ligand interaction profiler
RT: Retention time
TCA: Trichloroacetic acid
TMCS: Trimethylchlorosilane
